# Quantitative Viral Community DNA Analysis Reveals the Dominance of Single-Stranded DNA Viruses in Offshore Upper Bathyal Sediment from Tohoku, Japan

**DOI:** 10.3389/fmicb.2018.00075

**Published:** 2018-02-06

**Authors:** Mitsuhiro Yoshida, Tomohiro Mochizuki, Syun-Ichi Urayama, Yukari Yoshida-Takashima, Shinro Nishi, Miho Hirai, Hidetaka Nomaki, Yoshihiro Takaki, Takuro Nunoura, Ken Takai

**Affiliations:** ^1^Department of Subsurface Geobiological Analysis and Research, Japan Agency for Marine-Earth Science and Technology, Yokosuka, Japan; ^2^Project Team for Analyses of Changes in East Japan Marine Ecosystems, Japan Agency for Marine-Earth Science and Technology, Yokosuka, Japan; ^3^Earth-Life Science Institute, Tokyo Institute of Technology, Tokyo, Japan; ^4^Research and Development Center for Marine Biosciences, Japan Agency for Marine-Earth Science and Technology, Yokosuka, Japan; ^5^Department of Biogeochemistry, Japan Agency for Marine-Earth Science and Technology, Yokosuka, Japan

**Keywords:** deep-sea sediment, single-stranded DNA viruses, genome quantification, marine microbiology, metagenomics

## Abstract

Previous studies on marine environmental virology have primarily focused on double-stranded DNA (dsDNA) viruses; however, it has recently been suggested that single-stranded DNA (ssDNA) viruses are more abundant in marine ecosystems. In this study, we performed a quantitative viral community DNA analysis to estimate the relative abundance and composition of both ssDNA and dsDNA viruses in offshore upper bathyal sediment from Tohoku, Japan (water depth = 500 m). The estimated dsDNA viral abundance ranged from 3 × 10^6^ to 5 × 10^6^ genome copies per cm^3^ sediment, showing values similar to the range of fluorescence-based direct virus counts. In contrast, the estimated ssDNA viral abundance ranged from 1 × 10^8^ to 3 × 10^9^ genome copies per cm^3^ sediment, thus providing an estimation that the ssDNA viral populations represent 96.3–99.8% of the benthic total DNA viral assemblages. In the ssDNA viral metagenome, most of the identified viral sequences were associated with ssDNA viral families such as *Circoviridae* and *Microviridae*. The principle components analysis of the ssDNA viral sequence components from the sedimentary ssDNA viral metagenomic libraries found that the different depth viral communities at the study site all exhibited similar profiles compared with deep-sea sediment ones at other reference sites. Our results suggested that deep-sea benthic ssDNA viruses have been significantly underestimated by conventional direct virus counts and that their contributions to deep-sea benthic microbial mortality and geochemical cycles should be further addressed by such a new quantitative approach.

## Introduction

Viruses are abundant components in aquatic ecosystems and play important roles in microbial mortality, gene transfer, and biogeochemical cycles (Fuhrman, 1999; Wommack and Colwell, 2000; Suttle, 2007). For many years, most viral ecology studies have focused on double-stranded DNA (dsDNA) viruses that infect bacteria (bacteriophages; also known as phages) (Mann et al., 2003; Weinbauer, 2004). Recent viral metagenome (virome) studies have greatly expanded knowledge of marine dsDNA viruses, particularly their hidden diversity and global distribution (Brum and Sullivan, 2015). In addition, virome studies have also suggested a previously unseen diversity and co-occurrence of single-stranded DNA (ssDNA) viruses in the marine virosphere (Angly et al., 2006; Tucker et al., 2011; Labonté and Suttle, 2013; Yoshida et al., 2013). However, these studies have always performed whole-genome amplification by using the multiple displacement amplification (MDA) technique (Edwards and Rohwer, 2005). It has been noted that this technique has a substantial risk of preferential amplification of circular ssDNA to dsDNA, and thus, the relative abundance of ssDNA virome sequences and associated viruses may have been overestimated (Lizardi et al., 1998; Dean et al., 2001). To solve this issue, a less biased approach for DNA viral community analysis is required to quantify the relative abundance of viruses with different nucleic acid types in various environments. Recently, a new virome library preparation protocol using a commercial kit for both ssDNA and dsDNA viral templates has been developed to estimate the respective viral abundance on the basis of the metagenomic read abundance of relevant virus-associated sequences (Roux et al., 2016). Otherwise, a mass apportioning approach to viral nucleic acids can be used to estimate the abundance ratio of RNA and DNA viruses (Steward et al., 2013; Miranda et al., 2016); however, this approach has not been tested for ssDNA and dsDNA viral quantification.

Interactions between viruses and their hosts (e.g., bacteria and archaea) play important ecological and biogeochemical roles in deep-sea surface sediments (down to 50 cm below the seafloor [cmbsf]) (Danovaro et al., 2008a,b, 2016). Yoshida et al. (2013) have reported that the surface sediments from bathyal to hadal depths in the northwest Pacific harbor highly diverse ssDNA viromes, including sequences related to *Microviridae* phages and *Circoviridae* viruses. However, the abundance and diversity of ssDNA viruses in marine benthic environments have not been quantitatively evaluated. Here, we present a mass apportioning-based virus community DNA analysis to investigate the relative abundance and composition of ssDNA and dsDNA viral assemblages in deep-sea surface sediments (down to 15 cmbsf) off Tohoku, Japan (water depth = 500 m), which is located in the eutrophic northwest Pacific region, by using the quantification of ssDNA and dsDNA viral genomes and subsequent metagenomic analysis for each of the viral genome assemblages. Because we first separated the total sediment viral DNA into ssDNA and dsDNA components and then prepared and analyzed the respective sequencing libraries, this analysis would make it possible to mitigate the preferential amplification of ssDNA in the total DNA metagenomic sample preparation during the MDA reaction and obtain more reliable data in the DNA viral composition analysis, as well as independently, provide unbiased estimates in terms of both the total viral abundance.

## Materials and Methods

### Sediment Samples

Short sediment cores (24 cm in length) were obtained using a multiple corer offshore from the Tohoku region in Japan (station N-500: 39°15′ N, 142°13′ E; water depth of 500 m) during the Japan Agency for Marine-Earth Science and Technology (JAMSTEC) MR12-E02 Leg3 cruise of the R/V Mirai in March 2012 (Nomaki et al., 2016a). The sediment cores were sliced at 1–5 cm intervals as previously described (Nomaki et al., 2016a), and the subsamples were stored at -80°C. Among them, three sections (0–2, 5–8, and 10–15 cmbsf) were used for the following analyses.

### Direct Viral and Prokaryotic Cell Counts

To enumerate the viral and prokaryotic cell abundances, previously described direct cell and viral count methods were applied with minor modifications (Middelboe et al., 2011; Yanagawa et al., 2014). Briefly, approximately 2 cm^3^ of the frozen sediment was promptly suspended in 10 ml of modified SM buffer (50 mM Tris-HCl, pH 7.5; 10 mM MgSO_4_; 100 mM NaCl) containing 2% (v/v) formaldehyde in a 50-ml centrifuge tube. The slurry was shaken with a ShakeMaster Auto (BioMedical Science, Tokyo, Japan) for 1 min at maximum speed and then sonicated for 1 min with an ultrasonic homogenizer (UH-50; SMT company, Tokyo, Japan) to detach viral particles from sediment matrices (Middelboe et al., 2011). After centrifugation for 5 min at 700 *g*, microbial cells in the supernatant were filtered with a 0.2-μm pore size black polycarbonate filter (Advantec, Tokyo, Japan). For viral counts, viral particles in the filtrate were further filtered with a 0.02-μm pore size Anodisc filter (GE Healthcare, Piscataway, NJ, United States). Direct cell and viral counts were obtained with 5× SYBR Gold (Invitrogen, Eugene, OR, United States; Chen et al., 2001) for 15 min at dark, by using an Olympus BX53 epifluorescence microscope at a magnification of 1,500× (Olympus, Tokyo, Japan). Each filter was mounted on a glass slide with a phosphate-buffered saline-glycerol mixture (1:1) containing 0.1% (w/v) *p*-phenylenediamine (Sigma–Aldrich, St. Louis, MO, United States) to minimize fading (Noble and Fuhrman, 1998). Typically, 10–20 microscopic fields were randomly selected, and >200 viral particles and >200 prokaryotic cells were counted for each filter.

### Small Subunit (SSU) rRNA Gene Tag Sequencing

Environmental DNA was extracted from each sediment sample (approximately 5 g) by using a PowerMax Soil DNA Isolation kit (Mo Bio Lab, Carlsbad, CA, United States). SSU rRNA genes were amplified by LA Taq polymerase (Takara Bio, Kusatsu, Japan) using a universal primer set of U530F and U907R (Nunoura et al., 2012). The Illumina adaptor sequence (5′-ACA CTC TTT CCC TAC ACG ACG CTC TTC CGA TCT-3′; Illumina, San Diego, CA, United States) and Illumina Multiplexing PCR Primer 2.0 (5′-GTG ACT GGA GTT CAG ACG TGT GCT CTT CCG ATC T-3′; Illumina) were added at the 5′ ends of both primers (U530F and U907R). The PCR mixture contained LA Taq polymerase (final concentration of 0.1 U μl^-1^), 1 × GC Buffer I (Takara Bio), dNTPs (final concentration of 0.25 mM), each primer (final concentration of 0.2 mM), and template DNA. The PCR conditions were as follows: 25 to 35 cycles of 20 s at 96°C, 45 s at 52°C, and 1 min at 72°C. Library preparation and sequencing with Illumina MiSeq are described in Hirai et al. (2017). For the PCR cycle number of each sample, we used the minimum cycle number that provided enough amplified products for pyrosequencing based on the preliminary PCR amplification experiments using the same templates.

The paired-end reads of 16S rRNA gene tags were merged together using the program PEAR v0.9.10 (Zhang et al., 2014). Low-quality reads that included 3% or more nucleotides with low-quality values (quality score <30) were removed using the FASTX-Toolkit v0.0.14^1^. PCR primers were removed from the processed sequences using Cutadapt v1.10 (Martin, 2011). The resulting SSU rRNA gene tag libraries were analyzed with QIIME v1.9.1 pipelines (Caporaso et al., 2010). Briefly, the sequences flagged as chimeras were removed and were then clustered into OTUs by 97% sequence identity in each library. The taxonomic position of each OTU was automatically assigned on the basis of BLAST analysis, using the SILVA128 database (Quast et al., 2013) as a reference data set of SSU rRNA genes. The sequences closely related to the potential laboratory contaminants described above were omitted from further analyses.

### Viral DNA Extraction and Quantification of ssDNA and dsDNA

In this study, sediment viral fraction prepared for the DNA extraction and the subsequent virome analysis was also used as sample for electron microscopic observation. Thus, to avoid damage to viral structure by sonication, we adopted a conventional and versatile virus extraction method (Casas and Rohwer, 2007) for analyzing the viruses in sediment sample. Approximately 50 cm^3^ frozen sediment from each core section was immediately suspended in 125 ml of modified SM buffer and dispensed into 50-ml centrifuge tubes. The slurry was shaken for 1 min with a ShakeMaster Auto instrument at the maximum speed and centrifuged at 1,500 *g* for 2 min. The supernatant was then centrifuged at 8,000 *g* for 10 min. After collection of the supernatant, 125 ml of fresh modified SM buffer was added to the remaining sediments, and the extraction procedure was repeated. The resulting supernatants were pooled (a total of approximately 250 ml) and filtered with a 0.22-μm pore size filter (Millipore). Viral particles in the filtrates were collected by ultracentrifugation at 235,000 *g* (Type 45 Ti rotor, Optima L-90K ultracentrifuge; Beckman Coulter, Fullerton, CA, United States) for 3 h at 4°C. The pellet was suspended in TM buffer (50 mM Tris-HCl, pH 7.5; 10 mM MgCl_2_) and was subjected to cesium chloride density ultracentrifugation at 207,000 *g* (SW41Ti rotor, Optima L-90K ultracentrifuge; Beckman Coulter) for 48 h at 15°C (initial CsCl density of 0.45 g ml^-1^ in TM buffer). Then, a fraction (∼1.2 g ml^-1^) potentially containing small microbial cells that passed through 0.2-μm pore size filter (Kuhn et al., 2014) was removed. Finally, the virus particles were collected in a density range from 1.2 to 1.5 g ml^-1^ (Thurber et al., 2009; Holmfeldt et al., 2012). The viral fraction was dialyzed in fresh TM buffer for more than 3 h at 4°C, and was ultracentrifuged and collected again under the conditions described above. The pellet was suspended in SM buffer up to 200 μl and treated with DNase I (final concentration of 0.05 U μl^-1^; Roche, Mannheim, Germany) for 1 h at 37°C to remove naked extracellular DNA. The addition of EDTA solution (pH 8.0; final concentration of 0.5 M) terminated the reaction. Viral particles were lysed with SDS and proteinase K, and the viral DNA was obtained by the phenol-chloroform extraction and ethanol precipitation (Yoshida et al., 2008). RNA contaminants in the sample were digested with RNase A (final concentration of 0.5 μg μl^-1^; Invitrogen, Carlsbad, CA, United States). For the purification of ssDNA, a QIAprep Spin M13 kit (Qiagen, Hilden, Germany) was used. For the purification of dsDNA, ssDNA in the sample was digested with S1 nuclease (final concentration of 0.5 U μl^-1^; Takara Bio) at 37°C for 30 min, and the reaction mixture was processed with a QIAamp DNA mini kit (Qiagen). The purified ssDNA and dsDNA were quantified using a Qubit 2.0 Fluorometer (Life Technologies, Eugene, OR, United States) with a Qubit ssDNA assay kit (Life Technologies) and a Qubit dsDNA HS Assay kit (Life Technologies), respectively.

### Virome Composition Analysis

The viral ssDNA and dsDNA assemblages were amplified using a REPLI-g Mini Kit (Qiagen, Valencia, CA, United States) for 13 h and were purified by phenol/chloroform/isoamyl alcohol extraction. The amplified DNA products were digested with S1 nuclease at 37°C for 30 min and purified with phenol/chloroform/isoamyl alcohol extraction. Then, the amplified DNA was sheared using a Covaris S220 instrument (Covaris, Woburn, MA, United States) under the manufacturer’s recommended conditions for generating a 350-bp peak. Then, sequencing libraries were constructed using an Ion Plus Fragment Library Kit and an Ion Xpress Barcode Adaptors (Life Technologies, Carlsbad, CA, United States). Emulsion PCR was performed using an Ion PGM Hi-Q OT2 Kit (Life Technologies), and sequencing was performed using an Ion Torrent PGM with an Ion PGM Hi-Q Sequencing Kit and an Ion 318 Chip Kit V2 (Life Technologies). The raw sequence reads were processed using CLC Genomics Workbench ver. 8.0.1 (CLC Bio, Aarhus, Denmark) to trim low-quality reads (with the parameters of a minimum Phred score of 20 and a minimum read length of 50). The processed reads of each virome were subjected to BLASTx analysis (Altschul et al., 1997) against the NCBI GenBank non-redundant (nr) protein database. To identify the viral populations by the virome analysis, the RefseqVirus database including the complete virus genome sequences was also used in the BLASTx analysis. MEGAN (MEtaGenome Analyzer; version 4.70.4) was used to assign taxonomic groups of viruses and cellular organisms (bacteria, archaea, and eukaryota) to query sequences based on BLAST search (*E*-values>10^-5^) (Huson et al., 2011). The rRNA genes in viromes were also evaluated by BLAST analysis against SILVA123 database (Quast et al., 2013). Principal component analysis (PCA) for the comparison of the viromes in this study and other references was performed using the prcomp function in R.

### Transmission Electron Microscopy

In this study, sediment viral fraction prepared for the DNA extraction and the subsequent virome analysis was also used as sample for electron microscopic observation. Thus, to avoid damage to viral structure by sonication, we adopted a conventional and versatile virus extraction method (Casas and Rohwer, 2007) for analyzing the viruses in sediment sample. A portion of the viral fraction taken from the 10–15 cmbsf sediment sample was fixed using 1% (v/v) glutaraldehyde. An aliquot of the phage suspension was absorbed onto Formvar/carbon-coated copper grids, followed by staining with 2% (w/v) uranyl acetate and analysis using a TECNAI 20 transmission electron microscope (FEI, Tokyo, Japan) at 120 kV. Images were obtained using a CCD Eagle digital camera (FEI).

### Nucleotide Sequence Accession Numbers

The metagenomic data obtained by the multiple virome analyses and SSU rRNA gene tag sequencing of the deep-sea sediment samples have been submitted to the DDBJ Sequence Read Archive (DRA)^2^ under accession number DRA005987.

## Results and Discussion

### Characteristics of the Sediment Samples

**FIGURE 1:**
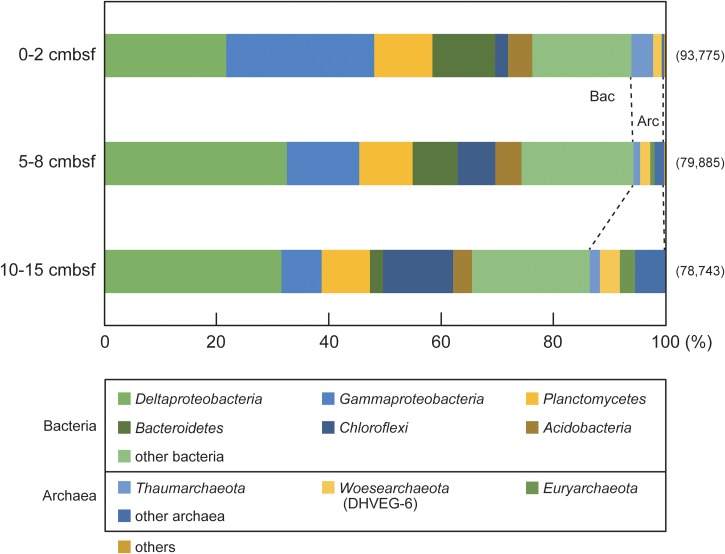
The depth profiles of prokaryotic community compositions based on the SSU rRNA gene sequences in deep-sea surface sediments from the off-Tohoku location (water depth = 500 m). The numbers of the sequences analyzed at each sediment depth are shown in parentheses. The “other bacteria” and “other archaea” categories represent the bacterial and archaeal taxa, respectively, and share less than 2% of the total sequences. The “others” category represents eukaryota and unclassified sequences.

Three sections (0–2, 5–8 and 10–15 cmbsf) of an upper bathyal sediment core (15 cm in total length) from the eutrophic northwest Pacific Ocean were used in this study to obtain a vertical profile of ssDNA and dsDNA viruses in deep-sea sediment (Nomaki et al., 2016a). The geochemical properties (Supplementary Figure S1) and biomasses in the sediment cores have been reported previously (Nomaki et al., 2016a,b). The direct virus and cell counts were reexamined for the virome samples analyzed in this study (**Table 1**), and they ranged from 5.1 × 10^5^ to 5.3 × 10^6^ particles per cm^3^ sediment and from 1.6 × 10^6^ to 9.1 × 10^6^ cells per cm^3^ sediment, respectively. The virus-to-cell ratio (VCR) decreased with increasing sediment depth, ranging from 0.32 to 0.58 (**Table 1**). The compositions of the small subunit (SSU) rRNA gene tag libraries are summarized in **Figure 1**. The predominant taxa were shared among the three sections, and their relative abundance changed along the sediment depth. The operational taxonomic units (OTUs) affiliated with *Gammaproteobacteria*, *Deltaproteobacteria*, and *Bacteroidetes* dominated the tag libraries in the 0–2 and 5–8 cmbsf sediments, whereas the *Deltaproteobacteria* and *Chloroflexi* OTUs were predominant at deeper depths (10–15 cmbsf). The archaeal OTUs belonging to *Thaumarchaeota*, *Woesearchaeota* (DHVEG-6), and *Euryarchaeota* were observed as minor components throughout all depths (3.8-8.1%).

**Table 1:** Direct cell and viral counts, the virus-to-cell ratio (VCR), the contents of viral DNAs and the estimated viral genome copy numbers of the ssDNA and dsDNA viral fractions, and the relative abundance of ssDNA viruses in the deep-sea surface sediments.

Depth (cmbsf)	Cells^a^ (cm^-3^ sediments)	Viral particles^a^ (cm^-3^ sediments)	VCR^b^	Content of viral DNAs (ng/cm^3^ sediments)		Estimated viral genome abundance (copies/cm^3^ sediments)		Relative abundance of ssDNA viruses (%)
				ssDNA	dsDNA	ssDNA^c^	dsDNA^d^	
0–2	(9.1 ± 1.1) × 10^6^	(5.3 ± 1.8) × 10^6^	0.58	2.87	0.29	(0.2–2.9) × 10^9^	5.3 × 10^6^	97.5–99.8
5–8	(5.9 ± 1.2) × 10^6^	(2.8 ± 0.2) × 10^6^	0.48	1.69	0.26	(0.1–1.7) × 10^9^	4.8 × 10^6^	96.3–99.7
10–15	(1.6 ± 0.9) × 10^6^	(5.1 ± 1.7) × 10^5^	0.32	1.50	0.17	(0.1–1.5) × 10^9^	3.0 × 10^6^	96.9–99.8

^a^*Data are presented as the mean ± SD for triplicate samples. ^b^Data are presented as the mean for triplicate samples. ^c^Ranges of the estimated genome copies for ssDNA viruses are calculated based on the ssDNA viral genome size range (1.8–24.9 kb) considering the latest virus taxonomy (www.expasy.org/viralzone) (Hulo et al., 2011). ^d^The estimated genome copies for dsDNA viruses are calculated based on the dsDNA viral average genome size (50 kb) reported by Steward et al. (2013)*.

### Construction and Evaluation of ssDNA and dsDNA Virome Libraries

Both ssDNA and dsDNA were extracted from viral particles in the sediment samples. The content of the viral ssDNA in the sediment samples ranged from 1.50 to 2.87 ng per cm^3^ sediment and was approximately one order of magnitude higher than those of the viral dsDNA (0.17–0.29 ng per cm^3^ sediment) (**Table 1**).

To examine the viral ssDNA and dsDNA assemblages, a total of six metagenomic libraries were constructed (**Table 2**). Totals of 1,091,418; 725,380; and 213,547 sequence reads were obtained from the ssDNA virome libraries from the 0–2, 5–8, and 10–15 cmbsf sediments, respectively. For the virome libraries prepared from the dsDNA viral fraction, totals of 751,300; 647,462; and 271,392 sequence reads were taken from the 0–2, 5–8, and 10–15 cmbsf sediment samples, respectively.

**Table 2:** Overview of quality-trimmed reads of the deep-sea sedimentary virome libraries analyzed in this study.

Sample depth (cmbsf)	Libraries	Number of reads	Average length (bp)	Total length (Mb)	GC content (%)	rRNA gene-associated reads
0–2	ssDNA	1,091,418	230.5	251	52.1	1
	dsDNA	751,300	226.6	170	61.1	0
5–8	ssDNA	725,380	222.2	161	54.5	0
	dsDNA	647,462	223.4	145	62.8	1
10–15	ssDNA	213,547	211.8	45	47.7	0
	dsDNA	271,392	218.1	59	52.7	0

**FIGURE 2:**
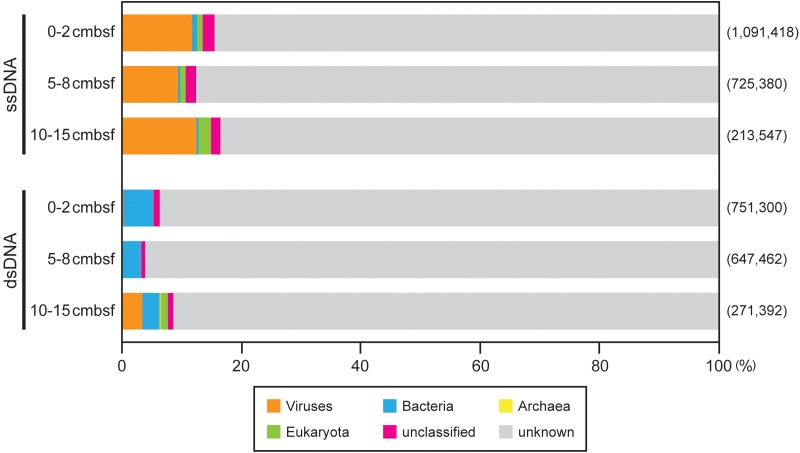
Summary of the MEGAN taxonomic assignments of the off-Tohoku sedimentary virome reads on the basis of BLASTx similarity search (*E*-value < 10^-5^) against the nr database. The numbers of the sequenced reads in each virome library are shown in parentheses.

To verify the quality of the viromes, their sequence reads were subjected to BLASTx analysis against the GenBank nr protein database as a reference data set of known genes (**Figure 2**). Only 4–17% of the sequence reads in each library showed significant similarity (*E*-value < 10^-5^ in BLASTx) to the sequences in the database and were subsequently classified into taxonomic groups by MEGAN analysis.

In the three ssDNA virome libraries, virus-associated reads were most abundant among the taxonomically assigned reads (9–13%), whereas more than 80% of each library exhibited no significant similarity to known sequences in the nr database (**Figure 2**). In contrast, in the dsDNA virome libraries, only 0.3–3% of sequence reads were classified as viral. The greater reads (3–5%) in these libraries showed significant homology to bacterial sequences, which was similar to the results of previous studies (e.g., Angly et al., 2006; Dinsdale et al., 2008). Most (75–94%) of the total annotated reads in each library were not assigned to any known KEGG metabolic functions (Kanehisa et al., 2012). Thus, the sequences assigned as non-viral are thought to be a result of the misclassification of viral sequences as host (bacteria, archaea, and eukaryota) genomic components, owing to both the lack of a comprehensive viral database and the horizontal gene transfer between viral and host genomes (Angly et al., 2006; Roux et al., 2012).

Among the reads classified as bacteria and archaea in each dsDNA virome library, the abundance of rRNA gene-associated reads was only 0–0.005% [for reference, length ratios of rRNA genes in genome sizes (1.5–5.5 Mb; Angly et al., 2009) inferred across aquatic bacterial and archaeal metagenomes: 0.03–0.1%], and it accounted for 0–0.0002% of the total virome reads (**Table 2**). According to the virome quality criteria proposed on the basis of the distribution of the rRNA gene-associated read ratios of the examined 67 public viromes (<0.02% of the total reads; Roux et al., 2013), our virome libraries produce relatively high quality with low contamination by cellular sequences.

### Composition of ssDNA and dsDNA Viromes

**FIGURE 3:**
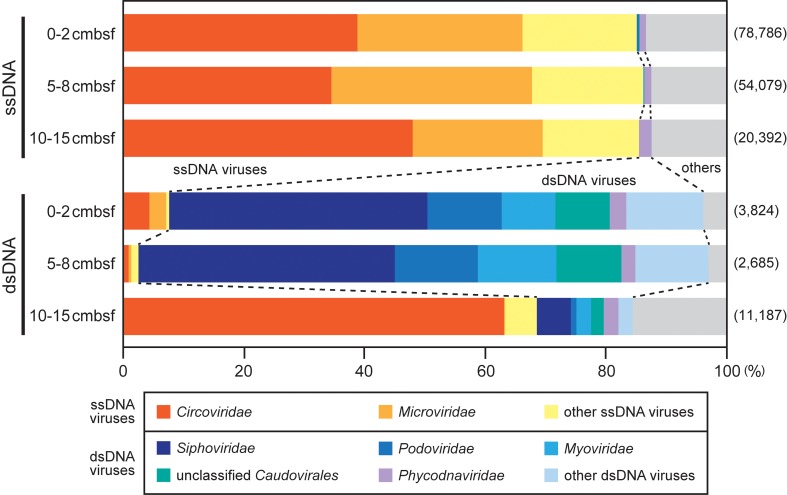
Summary of the MEGAN taxonomic assignments of viral reads in the off-Tohoku sedimentary virome libraries. The viral reads were identified by BLASTx similarity search (*E*-value < 10^-5^) against the viral sequence database constructed in this study. The numbers of the sequenced reads in each virome library are shown in parentheses. The “other ssDNA viruses” and “other dsDNA viruses” categories represent unclassified viruses and viral families constituting less than 2% of the total viral reads. The “others” comprises unclassified viruses and other components, single-stranded RNA viruses, retro-transcribing viruses, and virophages.

To examine the composition of ssDNA and dsDNA viromes, all reads of the sedimentary virome libraries (**Table 2**) were reevaluated by BLASTx similarity search (*E*-value < 10^-5^) against the RefseqVirus database including the complete virus genome sequences (**Figure 3**). Then, the reads that had significant similarity to the known viral sequences were defined as “viral reads” and were used for further analyses. The relative abundance of the viral reads in the ssDNA and dsDNA virome libraries ranged from 7.2 to 9.5% and from 0.4 to 4.1%, respectively.

In all the ssDNA virome libraries, most of the viral reads were related to genes from ssDNA viral families, which include *Circoviridae* and *Microviridae*, and they consisted of 85–86% of the total viral reads in each library (**Figure 3**). The family *Circoviridae* viruses and the marine relatives are known to infect eukaryotic hosts (animals and marine diatoms, respectively), whereas *Microviridae* is a bacteriophage group (e.g., Rosario et al., 2009; Tomaru et al., 2011). The sequences associated with these ssDNA viral families have also been found in other deep-sea surface sediments (Yoshida et al., 2013; Tangherlini et al., 2016), as well as in oceanic waters (e.g., Angly et al., 2006). The predominant *Inoviridae*-like sequences which predominated in the viral sequences from deep subseafloor metatranscriptomes (Engelhardt et al., 2015) were detected as minor components of the surface sediment ssDNA viromes. The reads originating from dsDNA viruses were rarely detected in the three ssDNA libraries (1–2%).

Among the dsDNA libraries, the viral read assemblages in the 0–2 and 5–8 cmbsf sections were dominated (88–94%) by sequences related to dsDNA viral families, which includes *Siphoviridae*, *Podoviridae*, *Myoviridae*, and *Phycodnaviridae* (**Figure 3**). The first three families are typical “tailed bacteriophages” belonging to the order *Caudovirales* and commonly occur in various marine and freshwater environments (Suttle, 2005; Roux et al., 2012), whereas *Phycodnaviridae* are aquatic viruses that infect eukaryotic algae (Larsen et al., 2008). The *Phycodnaviridae* viruses were detected as relatively minor populations in the dsDNA libraries (2.3–2.7% of the total viral reads). Because those families include viruses larger than 0.2 μm, some of them are likely to be selectively removed by filtration. The reads associated with ssDNA viruses ranged from only 3 to 8% of the total viral reads in the dsDNA libraries. In contrast to these dsDNA libraries, the relative abundance of the sequences associated with ssDNA viruses in the 10–15 cmbsf dsDNA library was unexpectedly greater than that associated with dsDNA viruses, sharing 69% of the total viral reads. This result was probably because of potential contamination of ssDNA due to the incomplete S1 nuclease digestion of ssDNA during the sediment viral dsDNA sample preparation. The overall composition of the virome libraries suggested that the viral ssDNA and dsDNA were generally well prepared from the sediment viral communities, although contamination of DNA types other than the target DNA type was not completely excluded from each DNA fraction sample.

**FIGURE 4:**
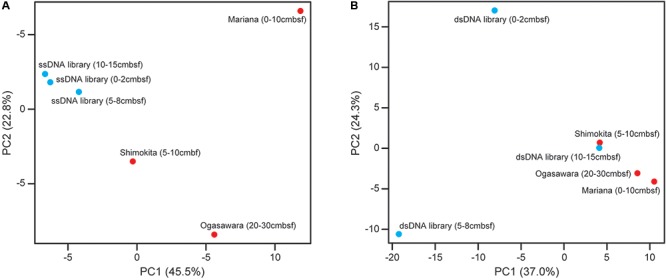
Principal component analysis (PCA) analysis of **(A)** ssDNA and **(B)** dsDNA viral sequence components in the relevant deep-sea sedimentary virome libraries from the off-Tohoku and other reference sites (Ogasawara Trench, Mariana Trench, and off Shimokita Peninsula) based on the taxonomic composition of viral communities. The respective off-Tohoku viral communities with the three different depths are shown in blue and the references are shown in red.

To examine the relationships among the different depth viromes in the present study and compare them with deep-sea sediment ones at other sites that we previously examined (Yoshida et al., 2013), the three data sets of the ssDNA and dsDNA viral sequence components in the relevant off-Tohoku sedimentary virome libraries (**Figure 3**) and the references were subjected to PCA (**Figure 4**). PCA for ssDNA viral components (**Figure 4A**) indicated that the different depth viral communities at the same site all exhibited similar profiles compared with those at other sites, while PCA for dsDNA viral components (**Figure 4B**) indicated that the different depth viral communities at the same site all revealed distinct relationships than with those at other sites.

### Abundance of ssDNA and dsDNA Viruses

The abundance of ssDNA and dsDNA viruses in the samples was estimated as the content of the viral ssDNA and dsDNA extracts (**Table 1**) divided by each DNA content per virus particle, which was calculated on the basis of the presumed genome sizes of currently known ssDNA and dsDNA viral families. The genome sizes of the ssDNA viruses, ranging from 1.8 to 24.9 kb, were determined according to the latest virus taxonomy on the ViralZone website^3^ (Hulo et al., 2011), and the DNA content per virus particle ranges from 9.90 × 10^-19^g (for 1.8 kb genome) to 1.37 × 10^-17^g (for 24.9 kb genome). The abundance of ssDNA viruses in the sediments of our study site ranged from 1.1 × 10^8^ to 2.9 × 10^9^ genome copies per cm^3^ sediment (**Table 1**). For the calculation of dsDNA viral abundance, we assumed an average DNA content per virus particle of 5.5 × 10^-17^ g (equivalent to 50 kb dsDNA) according to a previous estimate (Steward et al., 2013). The abundance of dsDNA viruses was estimated to be much lower than those of ssDNA viruses, ranging from 3.0 × 10^6^ to 5.3 × 10^6^ genome copies per cm^3^ sediment. Thus, we can provide estimation that the benthic ssDNA viral populations represent 96.3–99.8% of the benthic total DNA viral assemblages (**Table 1**). Further, morphological analysis of a sediment viral fraction (10-15 cmbsf sample) by transmission electron microscopy revealed the prevalence of non-tailed virus-like particles [diameter 31-62 nm (mean 47 nm), *n* = 20 particles] (e.g., Supplementary Figure S2) with sizes that were consistent with isolated marine ssDNA viral particles (diameter 30–73 nm; Brum et al., 2013), whereas there was no observation of tailed viruses, generally associated with dsDNA viruses. These data supported the results of the ssDNA and dsDNA viral quantification.

### Ecological Implications of ssDNA Viral Prevalence

Direct virus counting with SYBR staining is generally applicable to dsDNA viruses, whereas ssDNA and RNA viruses are often insufficiently stained, owing to their small genomes (Tomaru and Nagasaki, 2007; Tucker et al., 2011; Holmfeldt et al., 2012). In this study, the quantification of viral dsDNA and the subsequent calculation of viral abundance suggested that the estimated dsDNA viral abundances were comparable to the direct virus counts (**Table 1**). In contrast, the estimated ssDNA viral abundances were at least 200-fold higher than those obtained by direct virus count. The results suggested that the abundance of deep-sea sedimentary ssDNA viruses would have been extraordinarily underestimated by fluorescence-based direct counts. The benthic viral infection is responsible for the abatement of up to ∼80% of heterotrophic carbon production by bacteria and archaea, thus causing the release of ∼0.37 to 0.63 gigatons of carbon per year on a global scale (Danovaro et al., 2008b). Our quantitative approach to both ssDNA and dsDNA viral communities provides new insights into the re-evaluation of viral abundance and ecological functions in deep-sea sediments and illuminates the potentially significant contribution of ssDNA viral populations to the bacterial and archaeal mortality and biogeochemical processes of deep-sea benthic microbial communities by the viral shunt. Future work needs to determine whether other diverse marine benthic virosphere, including abyssal and trench sediments and deep subseafloor sediments, reveal the relatively high abundance of ssDNA viruses compared to that of dsDNA viruses.

## Author Contributions

MY conceived and designed the experiments, performed the experiments, analyzed the data, and wrote the manuscript. HN performed the onboard sample processing and critically edited the manuscript. TM, S-IU, and YY-T performed the laboratory experiments and critically edited the manuscript. MH performed the molecular analyses. SN and YT performed the bioinformatic analyses and critically edited the manuscript. TN and KT conceived and designed the experiments and wrote the manuscript.

## Conflict of Interest Statement

The authors declare that the research was conducted in the absence of any commercial or financial relationships that could be construed as a potential conflict of interest. The reviewer KW and handling Editor declared their shared affiliation.

## Footnotes

1 http://hannonlab.cshl.edu/fastx_toolkit/index.html

2 http://trace.ddbj.nig.ac.jp/dra/index_e.shtml

3 www.expasy.org/viralzone
