# Supplementary material for: Quantitative Viral Community DNA Analysis Reveals the Dominance of Single-Stranded DNA Viruses in Offshore Upper Bathyal Sediment from Tohoku, Japan
Source: Front Microbiol. 2018 Feb 6;9:75. doi: 10.3389/fmicb.2018.00075 (PMC5807898; doi:10.3389/fmicb.2018.00075)
Supplement: Supplementary file 1 [file Presentation_1.pdf]

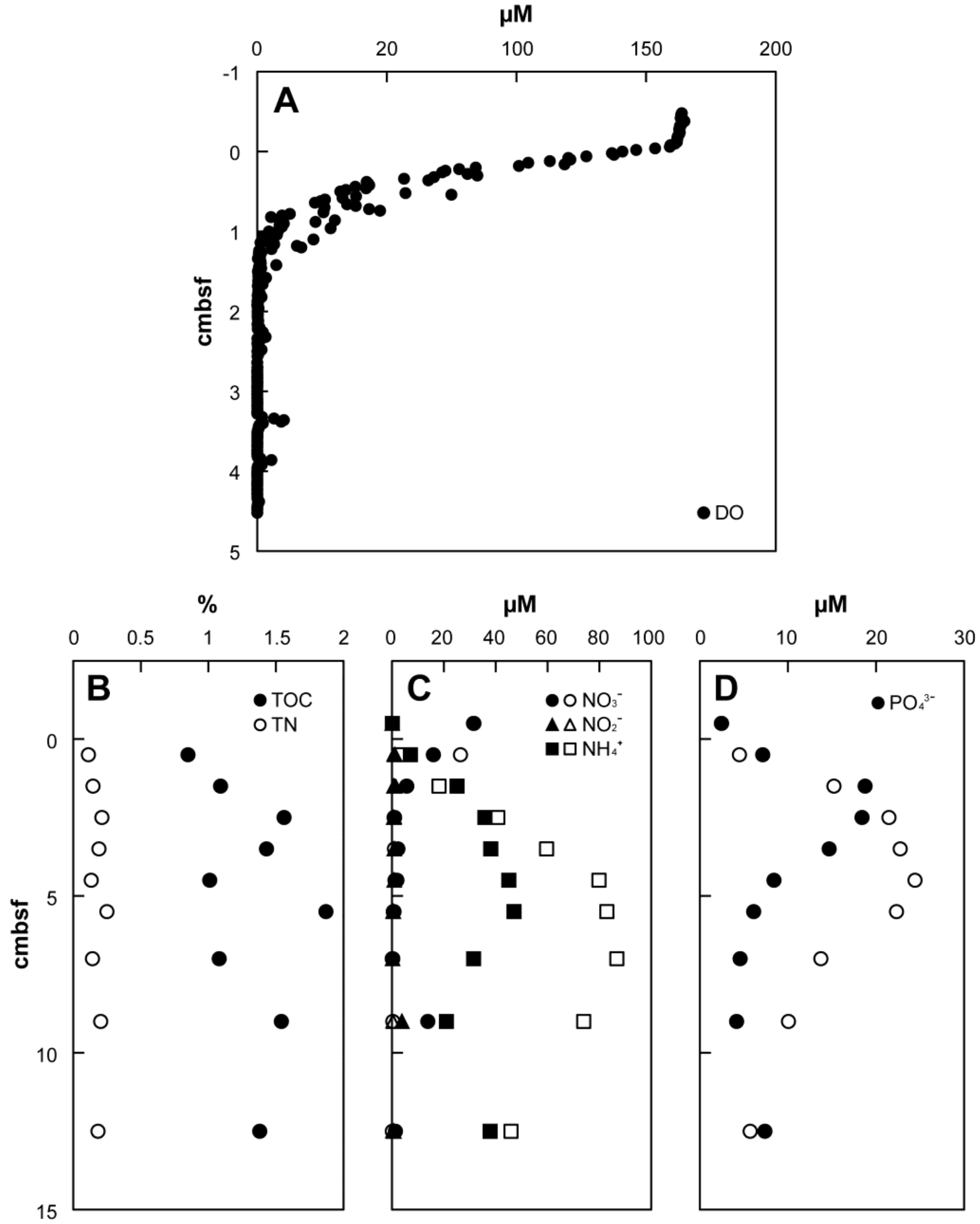

Figure S1

Geochemical characteristics of the off-Tohoku sediment core (station N-500 site; Nomaki et al., 2016a). (A) Dissolved oxygen concentration (μM). (B) Total organic carbon content (weight % of TOC) and total nitrogen content (wt% of TN). The TOC/TN ratios were constant (7.4–7.6) throughout the core depth. (C) Pore-water nitrate, nitrite, and ammonium and (d) phosphate concentrations (μM). Filled and open symbols represent profiles from replicate cores.

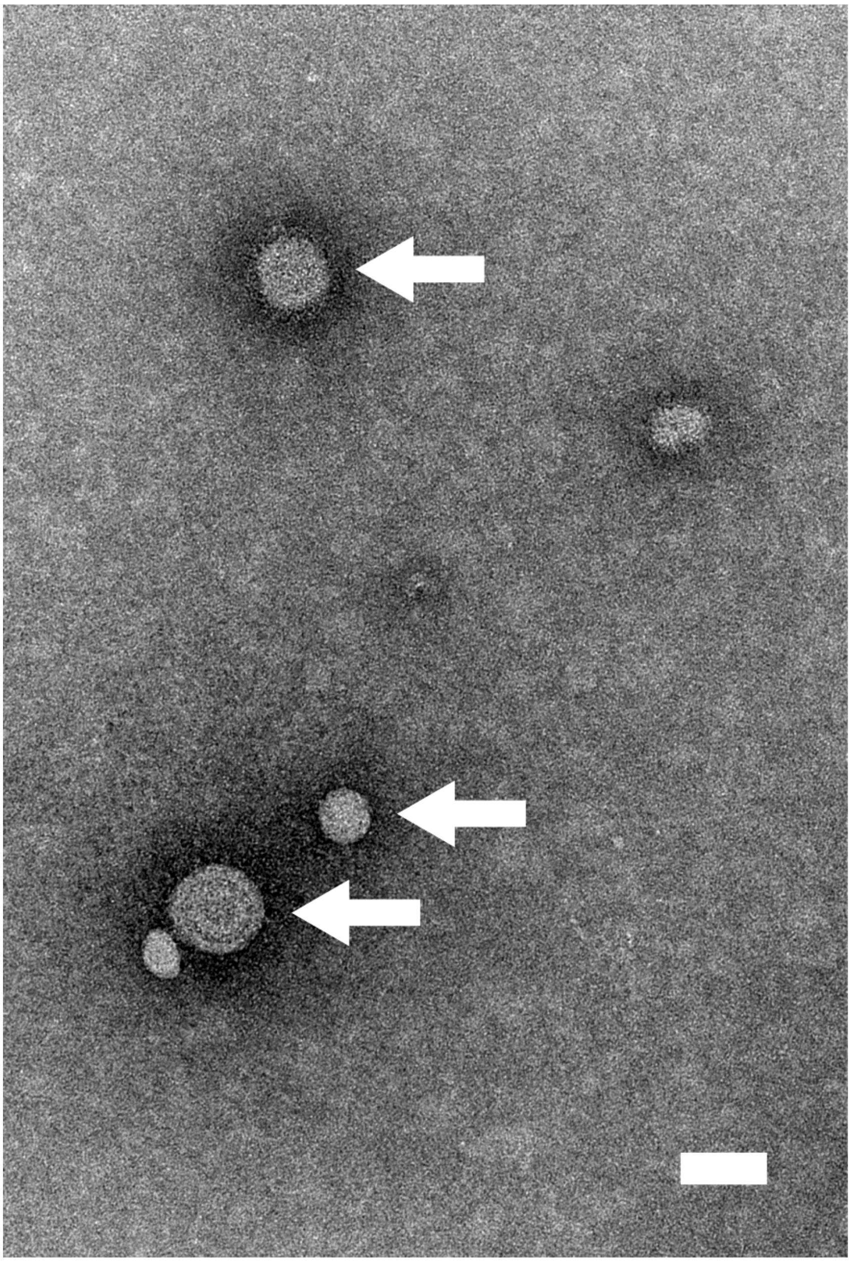

Figure S2

Transmission electron micrograph of non-tailed virus-like particles (arrows) observed in this study. The bar indicates 50 nm.
